# Tuberculous peritonitis in a case receiving continuous ambulatory peritoneal dialysis(CAPD) treatment

**DOI:** 10.1186/1476-0711-3-19

**Published:** 2004-10-04

**Authors:** Garip Sahin, Nuri Kiraz, Ilknur Sahin, Mehmet Soydan, Yurdanur Akgün

**Affiliations:** 1 Department of Nephrology, Osmangazi University Medical School, Eskisehir, Turkey; 2 Department of Microbiology, Osmangazi University Medical School, Eskisehir, Turkey; 3 Department of Chest Diseases, Osmangazi University Medical School, Eskisehir, Turkey

**Keywords:** CAPD, Tuberculosis peritonitis, Asid-fast basilli(AFB), Tuberculin skin test

## Abstract

**Background:**

Tuberculosis continues to be an important health problem in the world. Besides pulmonary involvement extrapulmonary involvement becomes an affair in developing countries, even in developed countries.

**Case presentation:**

A thirty-six year old male patient was admitted with abdominal pain, diarrhea, nausea, vomiting and fever which had started one week before. The patient had been followed up with predialisis Chronic Renal Failure(CRF) diagnosis for 4 years and receiving continuous ambulatory peritoneal dialysis (CAPD) treatment for 4 months. In peritoneal fluid, 1600/mm3 cells were detected and 70% of them were polymorphonuclear leukocytosis. The patient begun nonspesific antibiotherapy but no benefit was obtained after 12 days and peritoneal fluid bacterial cultures remained negative. Peritoneal smear was positive for Asid-fast basilli (AFB), and antituberculosis therapy was started with isoniazid, rifampicine, ethambutol and pyrazinamide. After 15 days his peritoneal fluid cell count was decreased and his symptoms were relieved. Peritoneal fluid tuberculosis culture was found positive.

**Conclusion:**

Considering this case, we think that in patients with CAPD catheter and peritonitis; when peritoneal fluid leukocytes are high and PMNL are dominant, AFB and tuberculosis culture must be investigated besides bacterial culture routinely.

## Introduction

Tuberculosis continues to be a devastatingly important health problem in the world. In addition to pulmonary involvement, extrapulmonary involvement becomes an issue in most developing countries. Extrapulmonary tuberculosis, because of several factors, has greatly contributed to the total tuberculosis mortalities during the 20^th ^century [1]. Risk of tuberculosis has increased due to decreased immunity in uremic patients. Tuberculosis comes out extrapulmonary with a rate of 40 percent in these patients, and periton is involved in 6 percent of all cases [2]. The risk increases in hemodialisis patients within 12 months after the beginning of treatment [3,4]. Tuberculous (TB) peritonitis is an event rarely seen in continuous ambulatory peritoneal dialysis CAPD patients [5]. Our case is presented as a rare TB peritonitis event receiving CAPD treatment.

## Case Report

The 36 year- old male patient, after receiving CAPD treatment for 4 months, consulted our clinic because of stomachache, diarrhea, nausea, vomiting and continous fever. The patient had been diagnosed with chronic renal deficiency and had been followed up with diagnosis of predialysis CRF for 4 years. The patient was referred to us because of his symptoms such as of nausea, vomiting, weakness, and a general condition of fatigue. Immediate care involved an urgent hemodialysis followed by CAPD and planning for renal replacement therapy.

Through a physical examination, the patient's blood pressure was 110/70 mmHg. The general condition was bad and pulmonary sounds in the respiratory system were diminished slightly in lower zones. On CAPD catheter's entering segment, infections were not seen.

In a laboratory investigation Hg was at 9.1gr/dl, Htc was at %27.6, WBC was at 5200/mm^3^, the platelet count was at 203000/mm^3^, and the erythrocyte sedimentation rate was at 22 mm/h. In biochemical findings, furthermore, serum creatinine was at 6.05 mgr/dl [ref. 0,5–1,4], urea nitrogen was at 38 mgr/dl [ref.5–20], protein was at 3.8gr/dl [ref:6–8.5], albumin was at 1.2 gr/dl [ref:3,5–5], and lactic dehidrogenase was at 565 iu/L. Serum sodium, potassium, glucose, bilirubin, alkaline phosphatase, aspartate and alanine aminotransferase, gamma-glutamyl transpeptidase, amylase, triglyseride, and cholesterol were normal. A coagulation factor protrombin time was found to be 18.7 sn. C-reactive protein was 9.54 mgr/dl. Bilateral costofrenic angles were blunted in posteroanterior pulmonary graphy.

No parasites and cystes were found in fecal examination due to diarrhea. No pathogenic agent was detected in stool cultures. In peritoneal cell counting, 1600/mm^3 ^cell were detected and it was seen that 70 percent of these cells were polymorphonuclear leukocytosis (PMNL). The patient was given ceftazidime (IV), cephazol, and amikacin (intraperitoneal), but no benefit was noticed after 12 days of antibiotherapy and there was no growth in peritoneal fluid cultures. There were PMNL present but no microorganism could be detected. Acid-fast basilli (AFB) was found to be positive in the gram staining of peritoneal fluid in the remaining follow up periods, and the patient had begun antituberculosis therapy in fours(with isoniazid, rifampin, ethambutol and pyrazinamide). Tuberculin skin test was anergical. On the 15^th ^day of anti tbc therapy, peritoneal fluid cell count decreased to 300/mm^3 ^. Peritoneal fluid bacterial culture, blood cultures, throat culture and urine culture were negative but peritoneal fluid tbc culture was found to be positive, in Lowenstein- Jensen medium in 24 days. The patient was followed up with the treatment for recovery with an anti-tbc treatment.

The peritoneal fluid of the patient was sent to be examined with Gram staining and Ziehl Neelsen staining. The peritoneal fluid was centrifuged at 3,000 × g for 15 minutes and the sediment was stained by Gram and Ziehl-Neelsen staining. The Gram staining showed PMNL presence but no microorganisms. The Ziehl-Neelsen staining(AFB) was positive.

The peritoneal fluid was transferred to 10 ml sterile glass tube and centrifuged at 3,000 × g for 15 minutes. The concentrated sediment was inoculated onto Lowenstein Jensen (LJ) medium without prior decontamination. LJ medium was incubated at 37°C. Two specimens were later sent to be examined with Ziehl Neelsen staining on two different days. Both of them were detected to be positive for Ziehl Neelsen staining.

LJ medium was examined for growth twice weekly for the first two weeks and once a week thereafter until the eighth week. After 24 days, the colonies were able to be seen on LJ medium. Positive growth was confirmed by Ziehl Neelsen staining.

## Discussion

CRF increases the risk of tuberculosis. In patients receiving hemodialisis, the risk of tbc increases within twelve months after the occurrence of extrapulmonary tbc. The risk in these patients is ten times more for extrapulmonary tbc than in any other population. Peritoneal tuberculosis is rarely seen but remains a very important complication in CAPD patients[5,6]. Mortality is high in these patients [7]. There are literatures showing mortality rates as high as 15 percent [8]. Quantrill at al., in a TB peritonitis study with 8 cases, found bacterial peritonitis as a source of the patient's complaints [5]. It was reported that this patient' acute course was atypical with a predominance of neutrophils and low levels of protein in the peritoneal fluid [9].

In English literature the most common complaints of tbc peritonit are as follows: fever (78 percent), stomachache (92 percent), misty dialisat (90 percent) and PMNL are dominant in peritoneal fluids in 76 percent of the cases and in 73 percent of the cases AFB and culture are positive [8].

Abraham et al. have reported tbc peritonit in 4 of 155 CAPD patients and tuberculin test were found anergical in all patients [10]. In our case the tuberculin test result was found anergical as well. In a retrospective study made by Lui et al. pulmonary or extrapulmonary tbc was detected in 38 of 790 CAPD patients and they obtained benefits on the 7^th^–57^th ^days of antituberculosis treatment (on average 30 day) [11]. We had experienced a recession in the peritonitis of the patient after 15th day of antituberculosis treatment.

It was reported in the literature; that in tbc peritonitis treatment, removing peritoneal catheter has no apparent benefit and does not increase efficacy of the treatment [2,6,9].

Considering this case, we think that in patients with CAPD catheter and peritonitis; when peritoneal fluid leukocytes are high and PMNL are dominant, AFB and tuberculosis culture must be routinely investigated along with bacterial culture.
